# Hemolytic anemia caused by an excessively kinked prosthetic graft after total arch replacement detected by 4-dimensional flow magnetic resonance imaging: A case report

**DOI:** 10.1097/MD.0000000000029617

**Published:** 2022-07-22

**Authors:** Yusuke Takei, Ikuko Shibasaki, Kohei Suzuki, Shohei Miyazaki, Shotaro Hirota, Hirotaka Ohashi, Shunsuke Saito, Hirotsugu Fukuda

**Affiliations:** a Department of Cardiac and Vascular Surgery, Dokkyo Medical University Hospital, Mibu-Machi, Tochigi, Japan; b Cardio Flow Design, Inc., Chiyoda, Tokyo, Japan.

**Keywords:** 4-dimensional flow magnetic resonance imaging, hemolytic anemia, viscous energy loss

## Abstract

Hemolytic anemia is a rare postoperative complication of aortic surgery, which may be caused by an excessively kinked graft that causes abnormal blood flow. It has been reported that 4-dimensional flow magnetic resonance imaging (4D flow MRI) can identify abnormal flow. Herein, we report the guidance of 4D flow MRI in performing the revision procedure for a patient with hemolytic anemia by evaluating abnormal blood flow based on this method.

**Patient concerns:**

A 70-year-old woman presented with dizziness and fatigue. She had undergone total arch replacement with a frozen elephant trunk 5 years prior. We diagnosed hemolytic anemia caused by a kinked graft after total arch replacement.

**Diagnosis:**

Although computed tomography findings revealed 3 lesions of the kinked graft at the ascending portion and cervical branches, 4D flow MRI findings showed that only the kinked graft at the ascending portion caused hemolytic anemia due to an elevated viscous energy loss around it.

**Intervention:**

We performed surgery to remove the kinked section instead of revision surgery consisting of total arch replacement.

**Outcomes:**

The patient’s postoperative course was uneventful and there were no complications. Postoperative enhanced computed tomography findings showed that the repaired graft had an adequate length and smoothly curved shape. The 4D flow MRI findings revealed smooth flow in the ascending portion and decreased viscous energy loss.

**Lessons:**

Based on the 4D flow MRI findings, we adopted a less invasive approach, repairing only the ascending portion of the graft, instead of performing revision surgery comprising total arch replacement.

## 1. Introduction

Hemolytic anemia after aortic surgery is a rare complication, especially by a kinked graft. One possible reason is that an excessively kinked graft may cause abnormal flow leading to a collision between the blood flow and the graft. The aforementioned results in conflicted blood flow, which causes energy loss, and the lost energy is consumed by destroying red blood cells.^[1,2]^ However, it is difficult to detect the flow by conventional imaging modalities. It has been reported that 4-dimensional flow magnetic resonance imaging (4D flow MRI) identifies abnormal flow, including quantitative assessments.^[3]^ Herein, we report the guidance of 4D flow MRI in performing the revision procedure for a patient with hemolytic anemia by evaluating abnormal blood flow based on this method.

## 2. Case report

**Figure 1.**
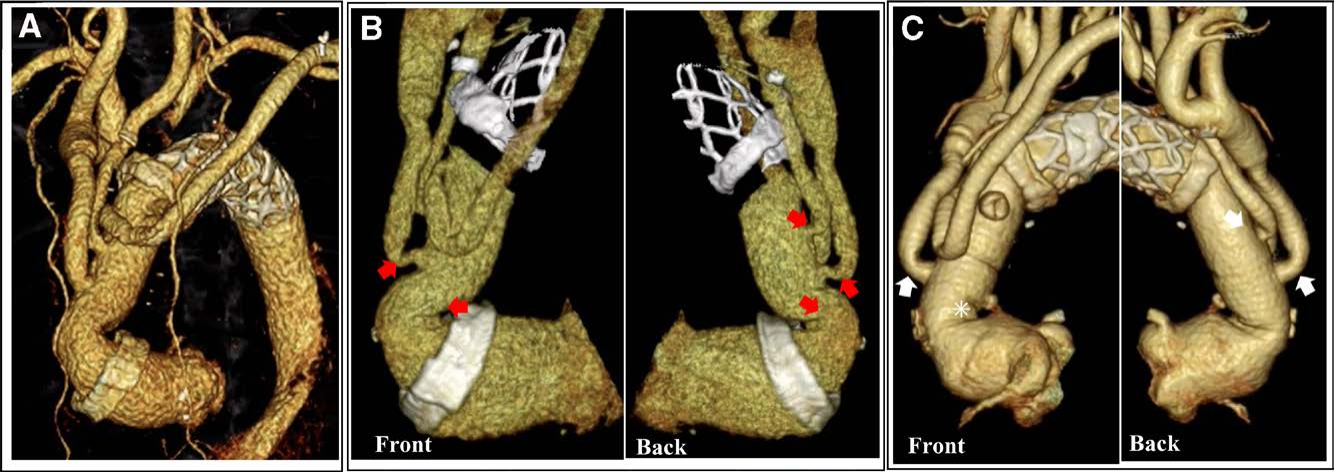
Pre- and postoperative CT images. (A) A volume-rendering image captured after the first surgery is presented. (B) An image taken before the revision surgery is presented. The red arrows show suspicious lesions of the kinked graft. (C) An image after the revision surgery is presented. The white asterisk shows the adequate length of the ascending portion of the graft. The white arrows show the result of the adequate angle of the first branch, but the second branch was unchanged. CT = computed tomography.

**Figure 2.**
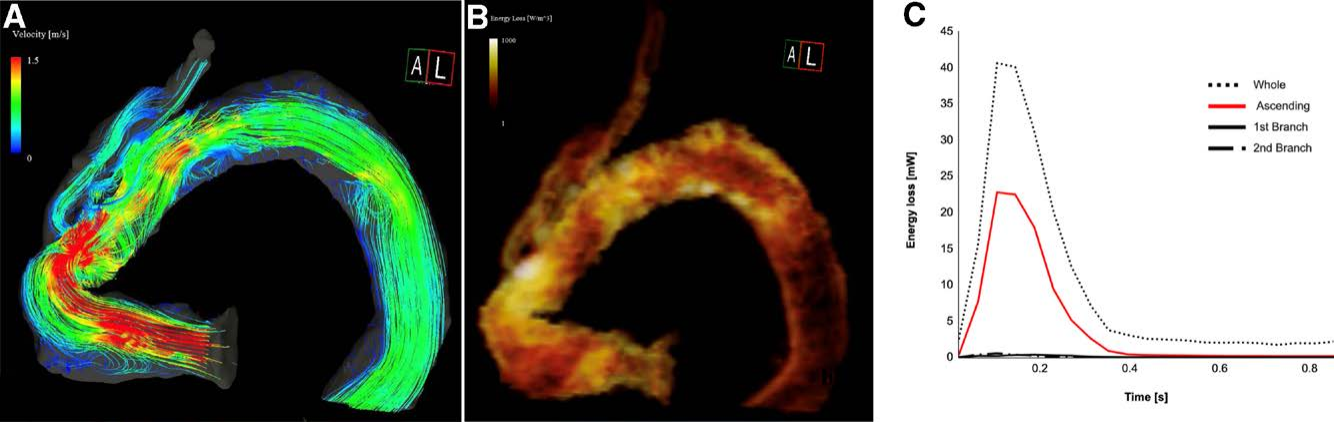
Four-dimensional flow MRI analysis of the streamlines and VEL before the revision surgery. (A) An acceleration flow is observed around the kinked lesion in the acceding portion of the graft in the streamlines. (B) The elevated VEL is represented by the bright area in the middle image. (C) The graph shows that the whole VEL was elevated. The VEL at the ascending portion was higher than that in the cervical branches. MRI = magnetic resonance imaging, VEL = viscous energy loss.

**Figure 3.**
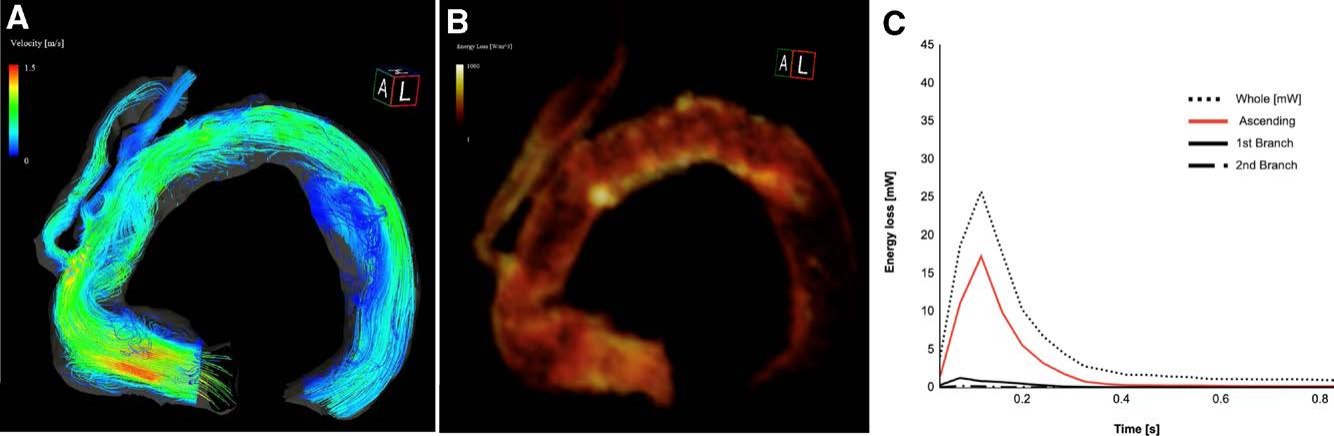
Four-dimensional flow MRI analysis of the streamlines and VEL after the revision surgery. (A, B) The acceleration flow and the bright area disappeared in the acceding portion of the graft. (C) The graph shows that the whole VEL and the VEL at the ascending portion decreased. MRI = magnetic resonance imaging, VEL = viscous energy loss.

A 70-year-old woman underwent total arch replacement with a frozen elephant trunk 5 years before using a previously reported technique for Stanford type A aortic dissection.^[4]^ The patient visited our office complaining of dizziness and general fatigue. After medical workup, the patient was finally diagnosed with hemolytic anemia. Laboratory findings revealed elevated lactate dehydrogenase enzyme levels (832 IU/L), elevated relative reticulocyte count (4.1%), and decreased haptoglobin levels (10 mg/dL). Schistocytes were detected in a peripheral blood smear, although Coombs’ test was negative, and there were no malignant cells in the bone marrow. Physical examination revealed a systolic ejection murmur at the aortic listening post. Transthoracic echocardiography revealed no evidence of valvular disease or left ventricular hypertrophy. Enhanced computed tomography (CT) findings showed a more sharply kinked graft in the mid-portion between the proximal anastomosis and the first branch of the 4-branched Dacron graft than after the first surgery (Fig. 1A, B). However, the scan did not reveal any endoleaks. In addition, the first and second cervical branches of the graft appeared to be slightly kinked at the origin. Therefore, we supposed that high-velocity and turbulent jet flow, caused by blood passing through those kinking narrow routes, induced mechanical damage to red blood cells. Furthermore, we performed a 4D flow MRI examination, which was approved by the institutional review board of our institute (Dokkyo Medical University), to evaluate and support our opinion. The 4D flow MRI examination was performed using a 3.0 Tesla MRI (Prisma fit 3T, Siemens AG, Munich, Germany) with respiratory motion compensation and electrocardiogram gating with full volumetric coverage of the thoracic aorta, without contrast media. The data from the acquired phase-contrast 3-axis cine images, magnitude images, and steady-state free procession cine images were analyzed with specialized software (iTFlow^®︎^2; Cardio Flow Design Inc., Tokyo, Japan).^[5]^ The streamlines representing the direction of the blood flow with flow acceleration demonstrated that highly accelerated vortex flow passed through the kinked graft at the ascending portion during the systolic phase; however, the same flow was not detected at the cervical branches that presented before they were kinked. The viscous energy loss (VEL) at the peak flow systole time point and the mean VEL per unit volume at the aortic arch were high, with a measurement of 40.6 mW (0.039 mW/mL). Measurements at the ascending portion were especially high at 22.8 mW (0.060 mW/mL), whereas those at the first and second branches were both low at 0.38 (0.007 mW/mL) and 0.56 mW (0.009 mW/mL), respectively (Fig. 2). Finally, based on the 4D flow MRI analysis findings, we concluded that mechanical hemolytic anemia was caused only by an excessively kinked graft in the ascending portion of the aorta. The patient’s symptoms gradually worsened, and she required repeated transfusions because her hemoglobin level was < 7.0 g/dL. Therefore, we scheduled surgery to remove the kinked section instead of performing a revision surgery consisting of total arch replacement. During revision surgery, a bent and kinked ascending aortic graft was observed. After aortic clamping, we opened the prosthetic graft to explore the proximal anastomosis and subsequently did not find any abnormal findings, such as stenosis of the proximal part of anastomosis for the Teflon felt strip, which was implanted to reinforce the anastomosis. Finally, we trimmed the graft, making it adequately shorter, and sewed up the graft with a continuous running suture. The patient’s postoperative course was uneventful and there were no complications. The postoperative enhanced CT findings showed that the repaired graft had an adequate length and smoothly curved shape (Fig. 1C), causing the kinked first branch to unexpectedly become positioned at an adequate angle, although the second branch remained unchanged. In addition, the 4D flow MRI findings revealed a smooth flow at the ascending portion, and a decreased VEL of 25.7 mW (0.024 mW/L) and 17.2 mW (0.033 mW/mL) at the aortic arch and the ascending aorta, respectively (Fig. 3). At the 6-month follow-up visit, the patient had recovered completely from hemolytic anemia and presented with no recurrence. The laboratory examination results revealed a hemoglobin level of 11.9 g/dL, lactate dehydrogenase enzyme level of 219 IU/L, relative reticulocyte count of 1.6%, and an increased haptoglobin level (248 mg/dL).

## 3. Discussion

Mechanical hemolytic anemia associated with aortic surgeries has been reported^[6]^ to have a considerably low prevalence. It occurs because of 2 main causes: anastomotic site stenosis with inverted internal felt strips and graft kinking. We described our experience with the latter, which causes different flow directions and leads to collision between the blood and the graft. Analysis with 4D flow MRI visually and quantitatively demonstrated this phenomenon using a streamline and VEL. VEL corresponds to the kinetic energy converted to thermal energy due to frictional forces induced by fluid viscosity.^[7]^ An elevated VEL represents the energy consumed for destroying blood cells, as observed in our case. This was reported for the first time by Uchida et al^[3]^ In our patient, the CT findings suggested that this patient’s hemolytic anemia was caused by the kinked graft in the ascending portion by the first and second branches of the graft. Based only on CT findings, we should have performed total arch replacement anew or considered performing catheterization to evaluate whether a pressure gradient existed at the lesions before revision surgery. Although we had performed catheterization, there was a possibility that we could not detect the pressure gradient because the graft was not stenosed but was kinked. Therefore, 4D flow MRI analysis revealed the generation of vortical flow and elevated VEL around the ascending portion of the graft. Moreover, this enabled us to decide to repair only the ascending portion of the graft instead of total arch replacement. It is emphasized that morphological assessments, such as those performed using CT, are insufficient, and we need to introduce functional assessment using this new modality.

However, this method has some limitations. First, VEL has been reported to be significantly elevated in patients with aortic valve stenosis.^[7]^ Second, the reference value of VEL in patients who underwent aortic surgery remains unclear, whereas that of healthy volunteers has been previously reported.^[7]^ Therefore, we evaluated the VEL along with streamlines in this patient. Further data on VEL in patients undergoing aortic surgery are needed to quantitatively assess VEL in the specific lesion where it occurs.

In conclusion, the 4D flow MRI findings demonstrated the cause of hemolytic anemia both visually and quantitatively in our patient. An increased VEL, based on the 4D flow MRI analysis results, detected where hemolytic anemia was mainly induced and guided us to perform less invasive revision surgery.

## Author contributions

**YT**: Conceptualization, data curation, investigation, methodology, draft writing, review writing, and editing. **IS**: Resources, review writing, and editing. **SM** and **KS:** Formal analysis, software, visualization, review writing, and editing. **SH** and **HO**: Resources. **SS** and **HF**: supervision, review writing, and editing. All authors have read and approved the final manuscript.

## Acknowledgments

We would like to thank Editage (www.editage.com) for English language editing.
